# Layer‐specific microstructural patterns of anterior hippocampus in Alzheimer's disease with ex vivo diffusion MRI at 14.1 T

**DOI:** 10.1002/hbm.26062

**Published:** 2022-09-02

**Authors:** Zhiyong Zhao, Lei Zhang, Wanrong Luo, Zuozhen Cao, Qinfeng Zhu, Xueqian Kong, Keqing Zhu, Jing Zhang, Dan Wu

**Affiliations:** ^1^ Key Laboratory for Biomedical Engineering of Ministry of Education, Department of Biomedical Engineering, College of Biomedical Engineering and Instrument Science Zhejiang University Hangzhou China; ^2^ China Brain Bank and Department of Neurology in Second Affiliated Hospital, Key Laboratory of Medical Neurobiology of Zhejiang Province, and Department of Neurobiology Zhejiang University School of Medicine Hangzhou China; ^3^ Department of Chemistry Zhejiang University Hangzhou China; ^4^ Department of Pathology, The First Affiliated Hospital and School of Medicine Zhejiang University Hangzhou China

**Keywords:** Alzheimer's disease, anterior hippocampus, connectivity, diffusion MRI, histology, mesoscale

## Abstract

High‐resolution ex vivo diffusion MRI (dMRI) can provide exquisite mesoscopic details and microstructural information of the human brain. Microstructural pattern of the anterior part of human hippocampus, however, has not been well elucidated with ex vivo dMRI, either in normal or disease conditions. The present study collected high‐resolution (0.1 mm isotropic) dMRI of post‐mortem anterior hippocampal tissues from four Alzheimer's diseases (AD), three primary age‐related tauopathy (PART), and three healthy control (HC) brains on a 14.1 T spectrometer. We evaluated how AD affected dMRI‐based microstructural features in different layers and subfields of anterior hippocampus. In the HC samples, we found higher anisotropy, lower diffusivity, and more streamlines in the layers within cornu ammonis (CA) than those within dentate gyrus (DG). Comparisons between disease groups showed that (1) anisotropy measurements in the CA layers of AD, especially stratum lacunosum (SL) and stratum radiatum (SR), had higher regional variability than the other two groups; (2) streamline density in the DG layers showed a gradually increased variance from HC to PART to AD; (3) AD also showed the higher variability in terms of inter‐layer connectivity than HC or PART. Moreover, voxelwise correlation analysis between the coregistered dMRI and histopathology images revealed significant correlations between dMRI measurements and the contents of amyloid beta (Aβ)/tau protein in specific layers of AD samples. These findings may reflect layer‐specific microstructural characteristics in different hippocampal subfields at the mesoscopic resolution, which were associated with protein deposition in the anterior hippocampus of AD patients.

## Graphical abstract

This study firstly and directly investigated the relation between microstructure in anterior hippocampus and the underlying pathology in a layer‐specific manner in Alzheimer's disease (AD). Our findings suggest that the molecular changes of Aβ and tau protein aggregations in AD may have a layer‐specific effect on the hippocampal microstructure.

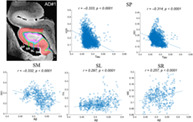

## 1 INTRODUCTION

Hippocampus is a complex and highly connected archeocortical structure in the brain, which can be divided into well‐defined subfields and layers with distinct morphological, molecular, electrophysiological and functional profiles (Kandel et al., 2014). Specifically, the hippocampal formation is composed of eight subfields, including cornu ammonis (CA1‐4), dentate gyrus (DG), fimbria, and the adjacent subiculum and presubiculum. Furthermore, CA1–CA3 consist of four layers**—**stratum pyramidale (SP), stratum oriens (SO), stratum lucidum (SL), and stratum radiatum (SR), while CA4 and DG are separated into three layers**—**stratum molecular (SM), stratum granular (SG), and polymorphic (PO) layers. Functionally, the hippocampus is involved in declarative memory and its damage has been associated with memory decline in several dementia‐related disorders (Kandel et al., 2014), such as Alzheimer's diseases (AD).

Multiple studies have reported hippocampal atrophy in AD patients (Barnes et al., 2004; Van et al., 2006), which presented subfield‐specific changes that the CA1 and subiculum are the most affected but the DG seems largely intact in AD (de Flores et al., 2015; Small, 2014). Alongside with neuroimaging results, histological studies in AD have showed that neurofibrillary tangles, neuronal loss, and decrease in synaptic density are dominant in the CA1, while the DG is relatively spared (Rössler et al., 2002; Scheff et al., 2007). Therefore, investigations towards the intra‐hippocampus variability are essential to better understand the pathological mechanism underlying AD. However, so far, few neuroimaging studies have focused on the hippocampal layers in AD due to the limited resolution (>1 mm) at the macroscopic resolution with in vivo MRI. For instance, the SG layer of the DG is only 0.2‐mm thick, hence requiring at least a resolution of 0.1 mm to distinguish it from the surrounding layers (Ly et al., 2020; Modo et al., 2016). High‐resolution ex vivo MRI at high fields provides the possibility to look into the laminar architectures (Ly et al., 2020), and it has been used to construct the hippocampal atlas of subfields and layers (Adler et al., 2018; Iglesias et al., 2015).

Diffusion MRI (dMRI) has shown to be useful to identify microstructural alterations in the hippocampal subfield or layer early in the course of neurological disorders (Crombe et al., 2018), for example, the fractional anisotropy (FA) and mean diffusivity (MD) from the commonly used diffusion tensor imaging (DTI) (Basser & Jones, 2002; Pierpaoli, 1996) can be used to quantify the structural integrity and restricted water diffusion in the tissue. In the previous in vivo studies, some of the researchers found decreased FA in the entire hippocampus of AD patients compared with normal control (NC) (Hong et al., 2013; Tang et al., 2016), whereas others observed insignificant changes in the FA (Lee et al., 2017; Mak et al., 2017). The inconsistences may be related to heterogeneous microstructures within the hippocampus. Recent ex vivo dMRI studies have characterized the DTI features of subfields and layers in middle hippocampus of mesial temporal lobe epilepsy (Ly et al., 2020). Ke et al. (2020) reported that inter‐hippocampal connectivity and FA in several layers were correlated with seizure frequency in patients with epilepsy. Despite the high‐sensitivity of DTI‐based indices, the tensor model cannot handle scenarios of branching or crossing fibers (Crombe et al., 2018), which is common in complex microstructures such as the hippocampus, for example, the axons of the pyramidal neurons run across the Shaffer collateral, Mossy fiber, and prefrontal pathway. High‐order dMRI models or model‐free approach, that is, the generalized q‐sampling imaging (GQI) (Yeh et al., 2010) can be used to resolve the crossing fibers. The GQI‐based metrics have shown good specificity and sensitivity for the evaluation of white matter integrity (Yeh et al., 2010).

In hippocampal formation, the anterior portion is dominated by CA1 and the subiculum, which are most attacked by AD (Deleon, 1999). Anterior hippocampus has a larger variance and more complex structure than the other parts (de Flores et al., 2020), making the segmentation of the subfields and layers challenging. Recent ex vivo dMRI studies of hippocampal layers all focused on the middle or posterior hippocampus, whereas the layer architectures in anterior part is less known. Also, layer‐specific change of the hippocampal microstructures in AD has not been reported to the best of our knowledge. Moreover, this study also involved primary age‐related tauopathy (PART), which is a type of tau pathology typically in the entorhinal cortex and hippocampus either without Aβ deposits (tau+/Aβ−) or with minimal Aβ deposits (Duyckaerts et al., 2015). Although AD and PART have similar features in neuronal tau deposits (Duyckaerts et al., 2015), they have different cognitive outcomes, overall morbidity and the age range of maximal vulnerability (Bell et al., 2019; Teylan et al., 2019). Whether PART pathology inevitably progresses to AD remains controversial (Duyckaerts et al., 2015; Jellinger et al., 2015). Our recent study has revealed a layer‐specific difference between PART and AD in the magnetic susceptibility of anterior hippocampus (Zhao et al., 2021). Here, we are interested in exploring whether such a difference exist in the microstructures of hippocampal layers. In the present study, we developed a high‐resolution dMRI acquisition procedure at an ultra‐high field of 14.1 T, and used DTI and GQI methods to decipher the architecture in the anterior hippocampus of the postmortem human brain. Then, we assessed hippocampal microstructure changes in AD samples, as compared to PART and healthy control (HC), and further performed MRI‐histological correlations using immunofluorescent staining for amyloid beta (Aβ) and tau protein.

## 2 MATERIALS AND METHODS

### 2.1 Sample preparation

Ten fresh brains (Table 1) were dissected within 8 h after death according to the Standardized Operational Protocol for Human Brain Bank in China (Qiu et al., 2018). Ethical approval was obtained for all experimental procedures from the ethics committee of Zhejiang University School of Medicine. All tissues were obtained under donor consent and provided by the National Health and Disease Human Brain Tissue Resource Center. Diagnosis of AD and PART was confirmed at autopsy, and the controls were selected from cases with no vascular or other neurological complications. The right hemisphere was fixed in 4% paraformaldehyde, and then dissected into small blocks. Next, the anterior hippocampal blocks (~5 mm thick block starting from the most anterior tip) were transferred to phosphate‐buffered saline (PBS) with 1 mM Gd‐DTPA (Berlex Imaging) for over 72 h. Finally, the specimens were placed in a 25‐mm diameter tube (which was filled with Fomblin) with supporting materials for stabilization during MRI scanning (Zhao et al., 2021).

**TABLE 1:** Summary of hippocampus samples

Sample	Age	Gender	Brain weight (g)	Fixation time (day)	Hemisphere
HC1	42	M	1300	25	Right
HC2	49	M	1270	24	Right
HC3	50	M	1430	25	Right
AD1	86	M	1360	20	Right
AD2	74	M	1224	44	Right
AD3	83	F	1210	36	Right
AD4	88	M	1234	14	Right
PART1	81	M	1176	11	Right
PART2	69	M	1120	42	Right
PART3	80	F	1190	19	Right

Abbreviations: AD, Alzheimer's disease; F, female; HC, healthy control with no significant neuropathology; M, male; PART, primary age‐related tauopathy.

### 2.2 MRI acquisition

All MRI scans were performed on a vertical 14.1 T Bruker spectrometer (Bruker Biospin) with a 30 mm diameter volume transmitter. Three‐dimensional (3D) multiple gradient echo (MGE) images were acquired with the following parameters: repetition time (TR) = 200 ms; echo times (TE) = 3.5, 7.0, 10.5, 14, 17.5, and 21 ms; flip angle = 12°; field of view (FOV) = 28 × 24 × 16 mm^3^; matrix size = 280 × 240 × 120; voxel size = 0.1 × 0.1 × 0.1 mm^3^; and averages = 2. Diffusion‐weighted MRI images were acquired using a house‐made 3D diffusion‐weighted gradient spin‐echo (DW‐GRASE) sequence (Wu et al., 2013) with the following parameters: TR = 800 ms; TE = 32 ms; FOV = 28 × 24 × 16 mm^3^; matrix size = 280 × 240 × 24; voxel size = 0.1 × 0.1 × 0.7 mm^3^; diffusion duration = 3.6 ms; diffusion separation = 15 ms; 3 images (b0) without diffusion weighting and 30 noncolinear diffusion directions per shell and two shells at *b*‐value of 3000 and 6000 s/mm^2^.

### 2.3 Histopathology

After the MRI scans, the ex vivo hippocampus tissues were transferred to PBS before being dispatched for histology, and then treated using automatic dehydrating machines and embedded in the paraffin. Subsequently, the tissues were sliced into 7 μm slices and baked at 60°C for 2 days, and further stored at room temperature. Immunohistochemistry staining protocol and information of all antibodies used in this study can be found in the Supplementary Material. The hippocampal immunohistochemical images were collected by microscope at 100× magnification.

The stained RGB images were first transformed into the grayscale images and normalized into 0–255. Then, the extent of Aβ and tau protein deposition were quantified by subtracting the intensity from the maximum possible grayscale intensity (O'Callaghan et al., 2017), and then normalized into 0–1 for voxelwise correlation with the MRI measurements.

### 2.4 Diffusion MRI preprocessing and tractography

dMRI data were first denoised using MRtrix3 (https://www.mrtrix.org/). Then, the images were reconstructed using tensor‐based reconstruction to obtain FA and MD in DSI Studio (http://www.dsistudio.labsolver.org). We also employed the GQI method (Yeh et al., 2010) in DSI Studio to obtain normalized quantitative anisotropy (nQA) and isotropic diffusion (ISO). Briefly, the nQA measures the anisotropy of restricted diffusion for each fiber population and is more robust to the partial volume of crossing fibers than FA, while ISO index represents diffusivity of the isotropic components including both CSF and nondirectional restricted diffusion (e.g., intra‐ or extra‐cellular diffusion). Finally, fiber tracking of the whole specimen was performed in DSI Studio utilizing a local multi‐direction deterministic Euler fiber‐tracking algorithm (Yeh et al., 2013), with QA cutoff of 0.02, angle of 60°, step size of 0.05 mm, minimum length = 0.2 mm, maximum length = 50 mm, smoothing = 0.2, Otsu threshold = 0.6, and thread count of 12, similar to those reported in a recent ex vivo hippocampus study (Ke et al., 2020). We calculated connectivity matrix based on the number of streamlines between each pair of layers.

### 2.5 Segmentation of hippocampal layer

**FIGURE 1:**
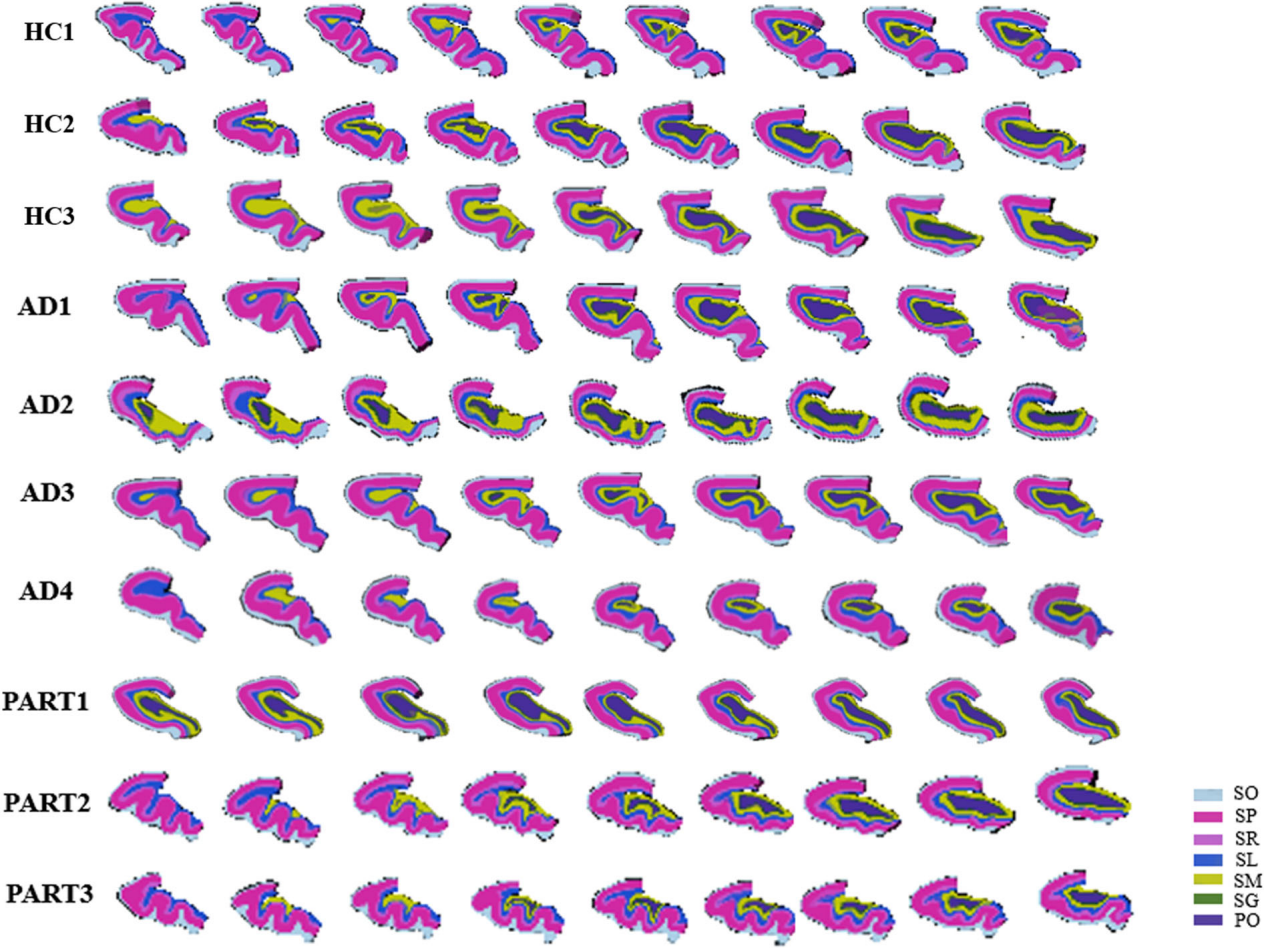
Cross‐sectional displays of anterior hippocampus segmentations for all samples in coronal view. PO, polymorphic layer; SG, stratum granulosum; SL, stratum lacunosum; SM, stratum moleculare; SO, stratum oriens; SP, stratum pyramidale; SR, stratum radiatum

Seven hippocampal layers, including four layers in CA and three layers in DG (Figure 1), were manually delineated by a trained technician (L.W.) and were confirmed by at least two team members, based on the second‐echo MGE images at TE = 7.0 ms that showed the best layer contrast among all anatomical images. The segmentation protocol was defined by neuroanatomists (Z.K. and Z.L.) and MR physicists (W.D. and Z.Z.), which was described in detail in our previous study (Zhao et al., 2021). Moreover, a reproducibility analysis showed that our manual segmentation had a very high inter‐ and intra‐rater reliability (Figure S4). Notably, the ROIs were drawn only for anterior hippocampal slices (~36 out of 120 slices for each sample). After that, the dMRI data (b0 image) were coregistered to the corresponding MGE images using linear registration in FSL (https://fsl.fmrib.ox.ac.uk/fsl/fslwiki/FLIRT), to extract the layer averaged values of FA, MD, nQA, ISO, and the total number of streamlines for each hippocampal layer. To account for volume differences, streamline density was also calculated as number of streamlines divided by the seed layer volume in the current study.

### 2.6 MRI‐histology co‐registration

In order to match the MRI and histological images of Aβ/tau at the voxel level for correlation analysis, we performed a slice‐to‐volume registration between the 2D histological image and the corresponding 3D MGE data for each sample (Figure S3). Specifically, we first identified the MGE slice which matched best with the histological section based on the second‐echo MGE image. Then, we performed a landmark‐based coarse registration, followed by intensity‐based affine and nonlinear transformations. More details can be found in our previous study (Zhao et al., 2021). Lastly, the dMRI data were registered to the MGE image, as described in the last section. The hippocampus was delineated manually in MR image and histology images independently before and after registration by an anatomist (Z.K.) (who are blinded for these images), respectively. The result showed a significantly higher similarity (dice index = 0.96) than that before registration (dice index = 0.62) (Figure S3c).

### 2.7 Statistical analysis

Considering the small sample size, no group comparison was performed in the current study. Instead, we compared the variability of the dMRI measurements among the three groups, as done in previous studies (Ke et al., 2020). The HC group was much younger than other two groups, and the volume of anterior hippocampus showed a significant negative correlation with age (*r* = −0.71, *p* = .02) across all samples in the present study. Thus, before and after correcting the age and the volume of anterior hippocampus by regressing them in a linear regression model, we calculated variability of the residuals for each dMRI measurement in each group, respectively. Moreover, Pearson correlations with the donors' age were calculated to determine how aging affects dMRI measurements. Finally, we performed the Pearson correlations between normalized Aβ/tau intensities and dMRI indices across the voxels in each layer for AD1, AD3, and PART2, as the staining quality for the other AD or PART samples was not sufficient for the correlation analysis (the staining images of AD2 and AD4 were severely damaged compared with the MR images; the hippocampal slice of PART1 is slightly folded in the corner, leading to a registration error between MRI and histology; and the histological section of PART3 was not included in the dissected hippocampal block for scan, Figure S2), and the control samples had minimal Aβ/tau deposition. All data analyses were performed in MATLAB 2019b (Mathworks Inc.).

## 3 RESULTS

### 3.1 Layer‐specific patterns of diffusional measurements in anterior hippocampus

**FIGURE 2:**
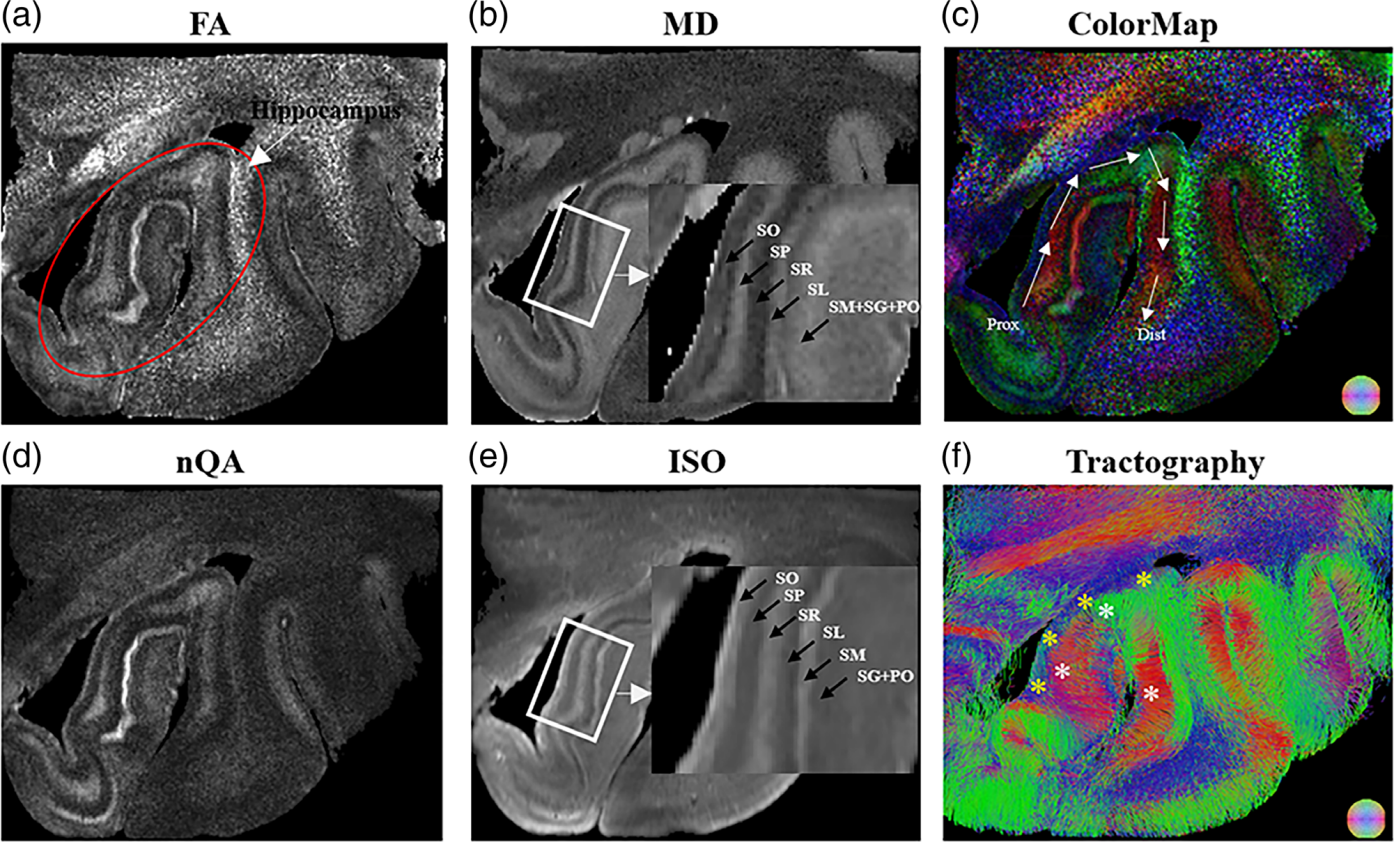
dMRI‐based microstructural maps and tractography of anterior hippocampus in a control sample. (a) and (b) represent the FA and MD maps from DTI analysis; (d) and (e) represent the nQA and ISO maps from GQI analysis, and zoom‐in view of the hippocampus (white rectangle) reveal the six hippocampal layers (black arrows); (c) and (f) show the directional encoded color maps and QA‐based tractography, respectively, where the white stars indicate the radial fibers that run perpendicular to the proximal‐distal axis (white arrows) and yellow stars indicate the fibers running along the proximal‐distal axis. DTI, diffusion tensor imaging; FA, fractional anisotropy; GQI, generalized q‐sampling imaging; ISO, isotropic diffusivity; MD, mean diffusivity; nQA, normalized quantitative anisotropy

Figure 2 presents the dMRI‐based microstructural maps and tractography in a HC case. Among them, the diffusivity maps (MD and ISO, Figure 2b,e) showed better contrasts between layers than the anisotropy indices (FA and nQA, Figure 2a,d), and the GQI‐based ISO maps identified more hippocampal layers than MD. For instance, both MD and ISO maps identified the boundaries between the four CA layers (SL, SR, SP, and SO); whereas the two DG layers were only distinguishable on ISO, but not MD. Moreover, the fiber bundle showed distinct orientations between the layers (Figure 2c,f). For example, the fiber tracts in SP and SM layers (white stars in Figure 2f) displayed a radial orientation perpendicular to the proximal‐distal axis (white arrows) (Figure 2c), possibly representing the well‐aligned axons of the hippocampal pyramidal cells, while, in the SO layer, fibers running along the proximal‐distal axis were observed (yellow stars) (Figure 2f).

**FIGURE 3:**
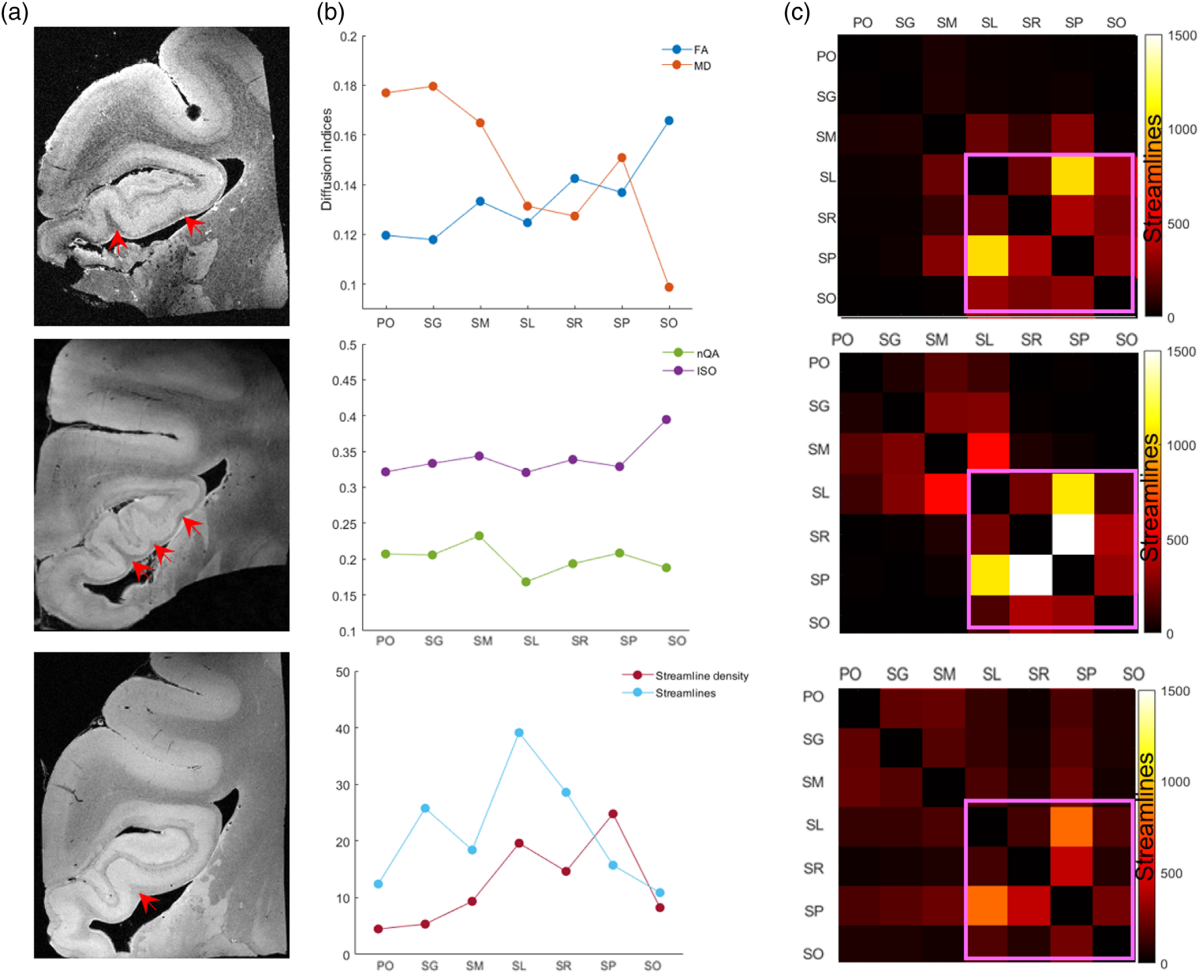
Layer‐specific pattern of the dMRI‐based microstructural indices and connectivity in three control samples. (a) the morphological differences in anterior hippocampus among three control samples. Red arrows point to the folding structures. (b) The dMRI indices, the number of streamlines, and streamline density averaged from the three control samples in each layer. (c) Structural connectivity matrix between the seven layers for each control case. The areas with pink rectangle indicated stronger connectivity among the CA layers than those among the DG layers or inter‐subfield connectivity (off‐diagonals). Streamline density = streamlines/volume/100. CA, cornu ammonis; DG, dentate gyrus; FA, fractional anisotropy; ISO, isotropic diffusivity; MD, mean diffusivity; nQA, normalized quantitative anisotropy

Quantitative analysis showed the three HC samples had individual differences in their morphology, for example, the number of folds in the anterior hippocampus (red arrows in Figure 3a) varied among them, although they shared similar layer‐specific dMRI patterns. The four layers within CA (SL, SR, SP, and SO) had higher FA and lower MD values than the three layers within DG (PO, SG, and SM), but nQA and ISO values did not show such differences (Figure 3b). Also, the four layers of CA contained more streamlines, with the SL and SP layers having the highest number of streamlines and streamline density within CA, respectively (Figure 3b).

Furthermore, three controls all displayed a stronger connectivity between the CA layers (Figure 3c, the areas with pink box) than that between the DG layers in the anterior hippocampus; and the inter‐subfield connections (off‐diagonals) were even weaker. Noticeably, the SL layer was heavily connected to all other layers in anterior hippocampus. Collectively, these findings reflected the layer‐ and subfield‐specific microstructural characteristics in the anterior hippocampus.

### 3.2 Intra‐hippocampal connections based on diffusion tensor imaging and generalized q‐sampling imaging

**FIGURE 4:**
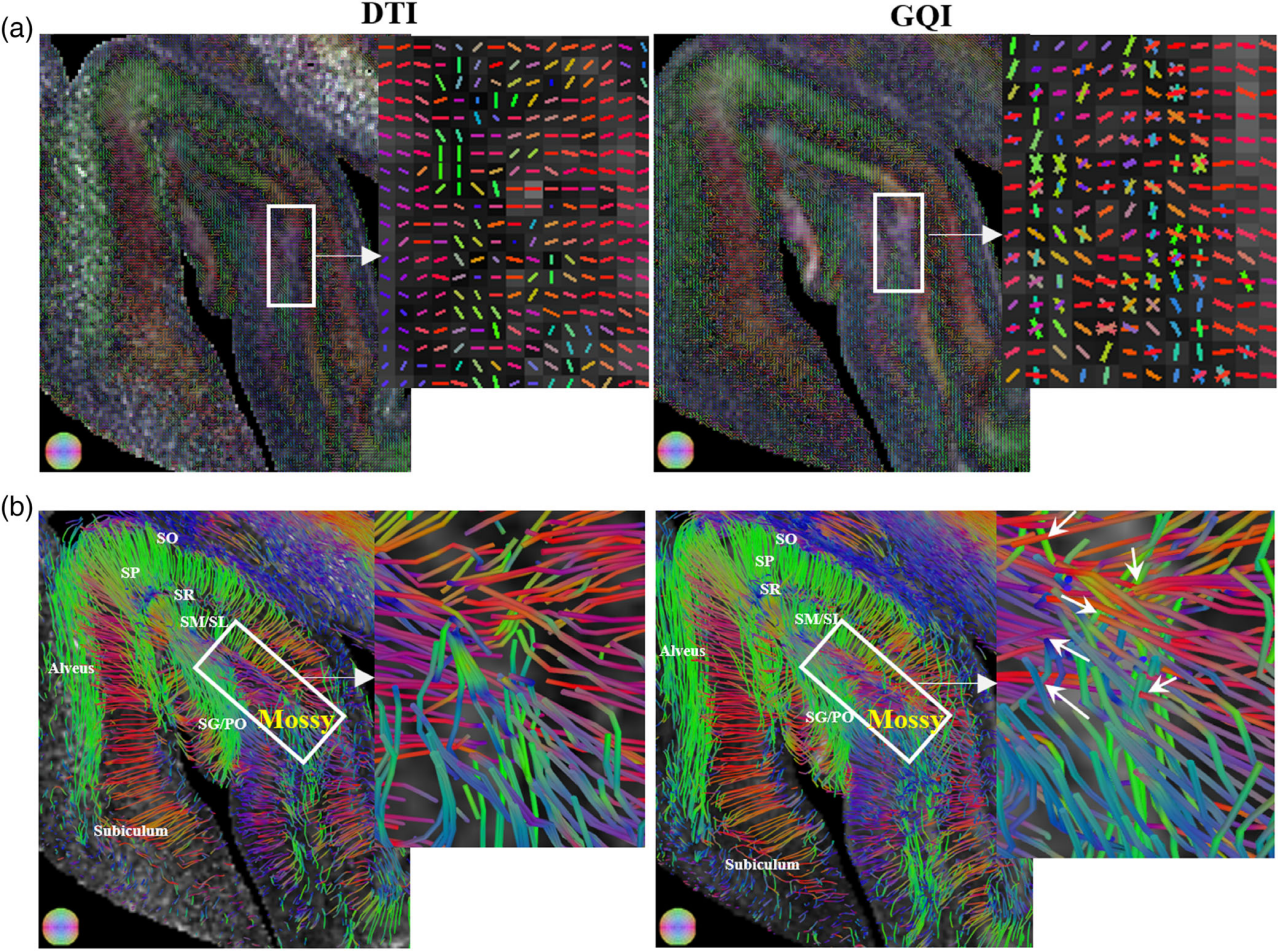
Comparison between DTI‐ and GQI‐based fiber orientation distribution maps (a) tractography (b) in anterior hippocampus of a control sample. The crossing fibers (white arrows) in hippocampal region (white rectangle) consist of Mossy fiber pathway (unmyelinated axons from granule cells in DG to modulatory hilar mossy cells in CA3), and the axons of hippocampal pyramidal neurons that run perpendicular to it. DG, dentate gyrus; DTI, diffusion tensor imaging; GQI, generalized q‐sampling imaging

Compared with DTI, GQI‐based tractography revealed more complex microstructures in anterior hippocampus. In the fiber orientation maps (Figure 4a), GQI identified the crossing fibers, corresponding to the radially running axons of pyramidal neurons and Mossy fibers that across them, which were not observed in DTI. Fiber streamlines reconstructed from the tensor and GQI methods highlighted intra‐hippocampal pathways (Figure 4b), such as the connections between granule cells in DG to CA3 (i.e., Mossy fibers, white rectangle).

### 3.3 The impact of Alzheimer's disease on hippocampal microstructure

**FIGURE 5:**
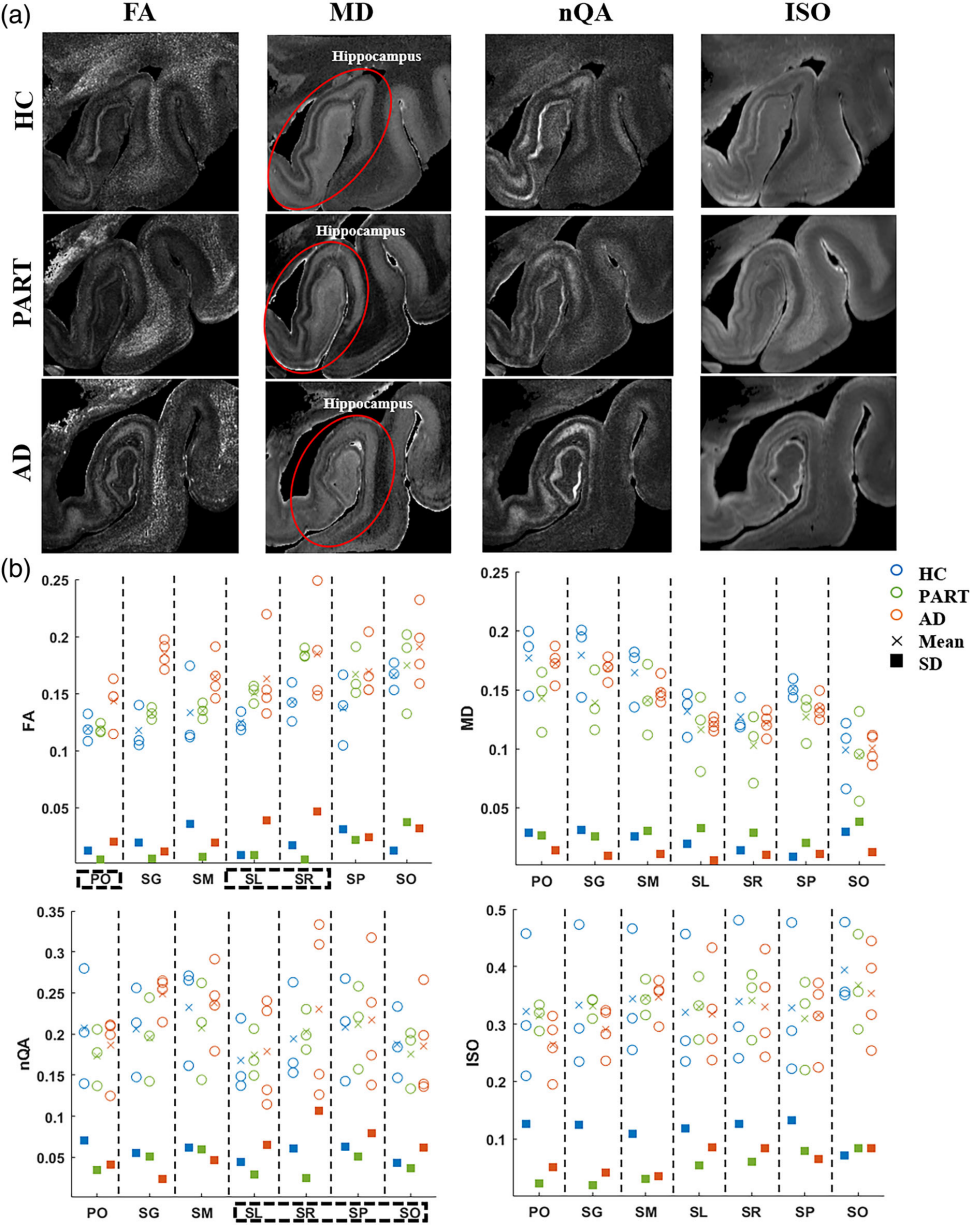
Comparisons of the dMRI‐based microstructural indices among three groups. (a) Coronal view of the FA, MD, nQA, and ISO maps of a control, PART and AD hippocampus. (b) Layer‐specific patterns of MD, FA, nQA, and ISO measurements in all samples. Variabilities of FA and nQA measurements were both higher in the SL and SR layers of the AD samples than those of HC and PART (black rectangle). Each circle represents an individual sample; blue, green, and red represent the HC, PART, and AD group, respectively; fork and square markers reflect the group mean and standard deviation, respectively. AD, Alzheimer's diseases; FA, fractional anisotropy; HC, healthy control; ISO, isotropic diffusivity; MD, mean diffusivity; nQA, normalized quantitative anisotropy; PART, primary age‐related tauopathy; SL, stratum lacunosum; SR, stratum radiatum

Figure 5a displayed dMRI‐based microstructural maps of anterior hippocampus in AD and PART specimen in comparison to HC. Quantitative analysis indicated AD‐related changes in several hippocampal layers (Figure 5b). Specially, compared to HC and PART, we found (1) FA values in AD group had higher variance in the PO, SL and SR layers and equivalent or lower variance in other layers; (2) MD values showed equivalent or lower variance in all layers; and (3) variability of nQA in AD group was higher in the four CA layers but lower in the three DG layers. Interestingly, ISO in HC group displayed the highest variance among three groups in all layers; while comparing PART and AD, the AD group had higher variance in PO, SG, SL, and SR layers.

**FIGURE 6:**
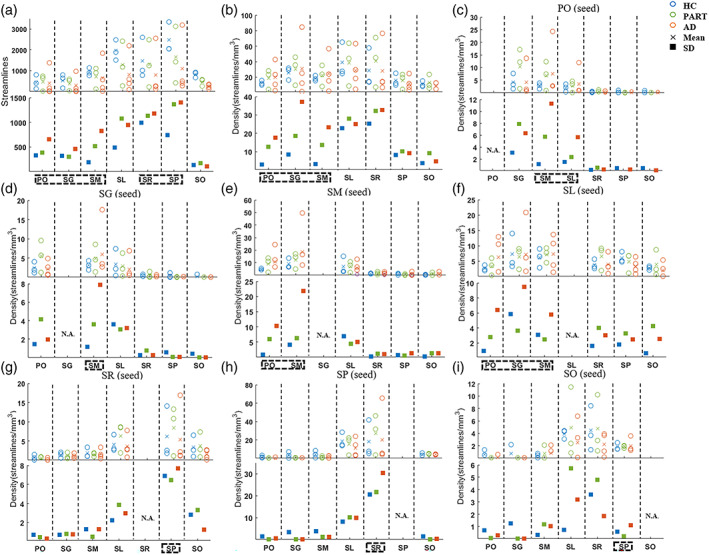
Layer‐specific patterns of the number of streamline and streamline density in each hippocampal layer (a, b) and the density from each of the seed layer to the other layers (c–i) for all samples. Variabilities were higher in the connections from three DG layers to the other layers of the AD samples than those of HC and PART (black rectangle). AD, Alzheimer's diseases; DG, dentate gyrus; HC, healthy control; PART, primary age‐related tauopathy

Tractography analysis revealed that the number of streamline and streamline density had the higher variance in the three DG layers of AD samples compared to PART and HC; while variance in the four CA layers was higher in AD than HC, but was equivalent to PART except for the SO layer (Figure 6a,b). Interestingly, we observed a gradually increased variance from HC to PART to AD in the streamline density in the DG layers (Figure 6b). Moreover, compared to PART and HC, AD also showed the highest variance in the connectivity between PO‐SM/SG‐SM (within DG), SR‐SP/SO‐SP (within CA), and PO‐SL/SL‐SG/SL‐SM (between DG and CA) (Figure 6c–i). In addition, the Pearson correlations analysis between the dMRI measures and the age indicated that aging had significant effects on microstructures of hippocampal layers (more details were displayed in Appendix S1). Both age and volume had no significant effect on the variability of dMRI measurements, the results after correcting the two factors were displayed in Figures S5 and S6.

### 3.4 Voxel‐wise correlation between diffusion MRI and histology

**FIGURE 7:**
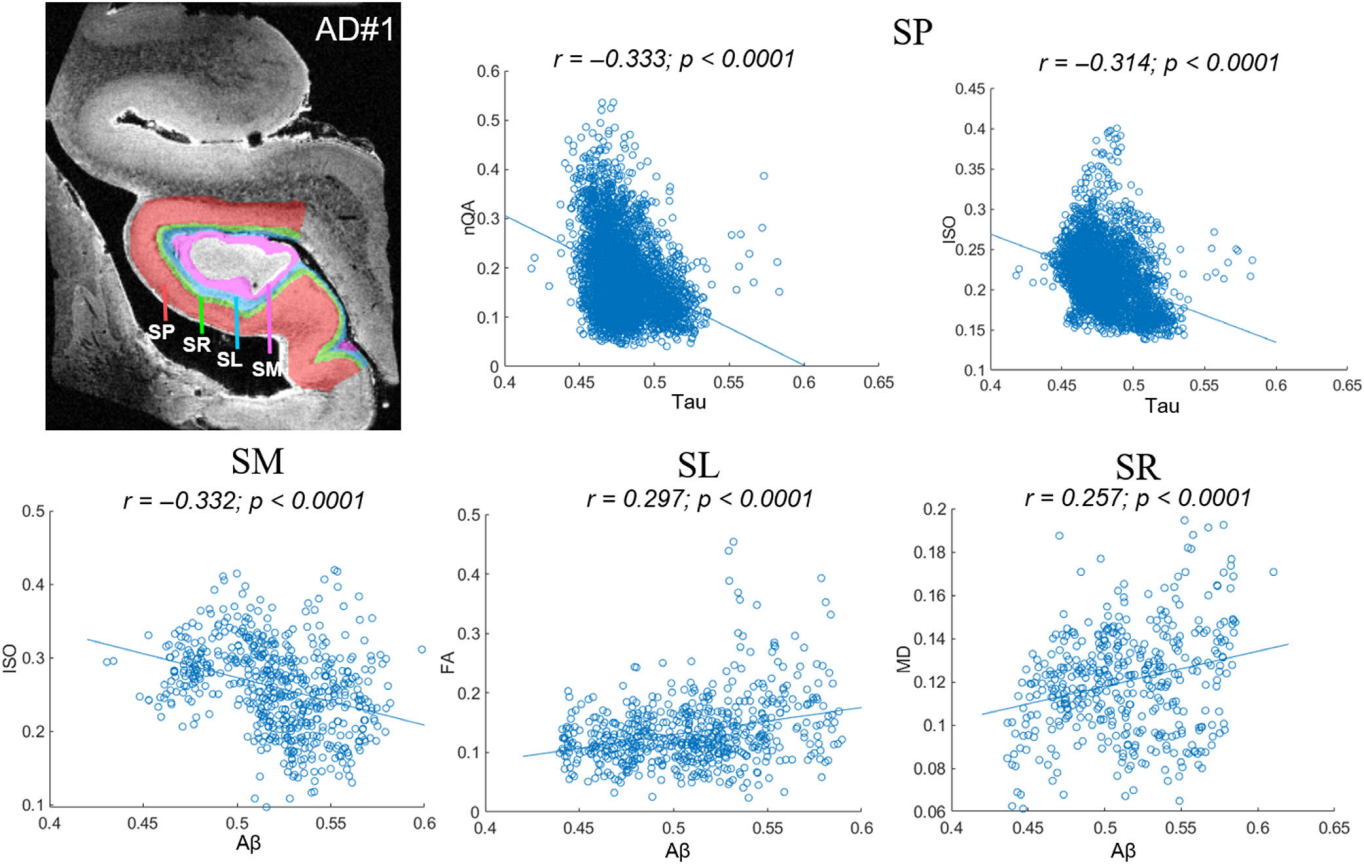
Voxel‐wise correlations between the normalized Aβ and tau intensities and the dMRI indices in AD1. Only the layers showing significant histology‐MRI correlations (*p* < .001) were plotted. AD, Alzheimer's disease; ISO, isotropic diffusion; FA, fractional anisotropy; MD, mean diffusion; nQA, normalized quantitative anisotropy; SL, stratum lacunosum; SM, stratum moleculare; SP, stratum pyramidale; SR, stratum radiatum

dMRI‐histology correlation analysis in AD1 sample showed significant positive correlations between Aβ and FA in SL layer and between Aβ and MD in SR layer, and a negative correlation between Aβ and ISO in SM layer (FDR adjusted *p* < .05, Figure 7). Moreover, the tau content in SP layer was negatively correlated with the nQA and ISO (adjusted *p* < .05) (Figure 7). We did not find significant correlations between the Tau content and the dMRI measurements in any of the hippocampal layers of AD3 and PART2.

## 4 DISCUSSION

In this study, we segmented the anterior hippocampus into the seven layers with high‐resolution ex vivo MRI, and then characterized and compared the layer‐specific microstructural patterns in HC, PART, and AD samples. We found that (1) model‐free GQI was able to resolve the complex microstructures and connections within the anterior hippocampus; (2) the variance of dMRI measurements in hippocampal layers was generally higher in AD than HC or PART; and (3) the accumulations of Aβ in the SM/SL/SR layers and tau in the SP layer were significantly correlated with the dMRI metrics in AD. These findings suggest that the molecular changes of Aβ and tau protein aggregations in AD may have a layer‐specific effect on the hippocampal microstructure.

### 4.1 Characteristics tissue microstructure in anterior hippocampus

Previous ex vivo studies in hippocampus mostly focused on the hippocampal body or middle hippocampus (Ke et al., 2020; Ly et al., 2020), which has a dark band with a “C” or reversed “C” shape in T2‐weighted image as a hallmark for segmenting the subfields and layers (de Flores et al., 2020). The anterior part of hippocampus is more complex with more folds and larger individual variances than the middle part (de Flores et al., 2020; DeKraker et al., 2020). Although several studies based on histological staining (Ding & Van Hoesen., 2015) and morphological measurement (de Flores et al., 2020) have depicted the anatomical details of the anterior hippocampus, few studies investigated its microstructural characteristics. Moreover, it is well known that the pyramidal neurons of the CA1 in the anterior hippocampus were the mostly easily attacked by AD process (Deleon, 1999). Therefore, identifying the microstructural pattern in anterior hippocampus may help to understand the pathological mechanism of AD.

The diffusivity maps exhibited excellent laminar contrasts as these laminas are composed of different cellular substrates (Bartsch & Wulff, 2015). For instance, the higher ISO in the SO, SR, and SM layers, where the axons and dendrites reside; while lower ISO in SP, SL, and SG/PO was possibly related to the densely packed pyramidal neurons and granule cells (Ding & Van Hoesen, 2015; Van Hoesen & Hyman, 1990). Quantitative analysis of the dMRI metrics showed layer‐specific differences that the four CA layers had higher FA and lower MD values than the DG layers in three control samples, but neither nQA nor ISO showed such pattern, possibly indicating that although the overall anisotropy was higher in CA than DG, the microscopic anisotropy considering all crossing fibers remained similar. Also, the higher inter‐layer connections in CA than DG possibly reflected contributions of the perforant path and Schaffer collateral (Beaujoin et al., 2018; Zeineh et al., 2017) that pass through the layers within CA.

### 4.2 Increased variances of the microstructure in hippocampal layers of Alzheimer's disease

A recent ex vivo study in hippocampal body using DTI found that patients with mesial temporal lobe epilepsy exhibited a larger variance in dMRI measures in hippocampal layers compared to controls (Ke et al., 2020). Authors explained that this difference might reflect the variability in clinical measures (i.e., seizure severity or frequency). Similarly, the present study found that AD specimen showed the largest variability in FA and nQA among three groups in the CA layers, likely associated with the differential AD pathology involved in different subfields of the hippocampus. These heterogeneous between AD patients may explain the inconsistent findings in previous in vivo AD studies that reported decrease or no change in FA (Lee et al., 2017; Tang et al., 2016). Previous ex vivo study of hippocampus found higher variance in magnetic susceptibility measurements in AD than controls that may be caused by the artifacts resulting from substantial cell damage in the hippocampus of AD (Antharam et al., 2012). Considering that FA primarily reflect the structural integrity (Chang et al., 2017) and nQA is associated with cellular density (Yeh et al., 2017) and axonal density (Garic et al., 2020), the large variances in nQA and FA may reflect AD‐induced microstructural changes, including decreased myelinated fibers (Lu et al., 2016), axonal damage (Sanchez‐Varo et al., 2012), and cellular loss (Jansen et al., 1993) in the anterior hippocampus.

Moreover, although it has been debated whether PART will eventually develop into AD (Duyckaerts et al., 2015; Jellinger et al., 2015), PART have different clinical and pathological features from AD. Evidence demonstrates that PART is a pathologic substrate for elderly individuals with memory impairments (Nelson et al., 2016), and the higher stage PART cases (Braak NFT stages III/IV) tend to show more severe cognitive impairments (Crary et al., 2014). A recent review suggests that PART at least represents a subtype of AD that has a later onset of symptoms with a slower rate of disease progression (Hickman et al., 2020). Our recent study found that the heterogeneity of magnetic susceptibility were higher in hippocampal layers of AD patients compared with the age‐ and gender‐matched PART cases, and the T2* values in the SR layer were correlated with the tau content in the PART but not AD (Zhao et al., 2021). Here, we found the variances in the connections from the three DG layers to the other layers and connections between PO‐SM/SL, SG‐SM, and SP‐SR layers gradually increased from HC to PART to AD, indicating the intra‐hippocampal connectivity changes may be an indicator of the pathological degeneration from PART to AD.

### 4.3 Relationship between histological and diffusional measurements

It has been proposed that the depositions of Aβ and tau plaques in AD may induce a toxic environment, which results in the changes of hippocampal microstructures, such as a decrease in the number of neurons, as well as changes in the morphology of the dendritic arbor and spines (Furcila et al., 2019). Several in vivo studies have used dMRI to examine the effect of Aβ and tau burden on hippocampal microstructures. For instance, studies found that higher Aβ burden was related to lower FA in the fornix (a whiter matter region in hippocampal formation) (Chao et al., 2013; Gold et al., 2014); higher CSF T‐Tau/Aβ42 was associated with higher diffusivity in the left temporal lobe (Bendlin et al., 2012). Snow et al. (2017) also found decreased FA in the hippocampal gray matter regions with both congophilic Aβ plaques and tau accumulation. Zhou and Bai (2017) reported voxel‐wise negative correlations between Tau and FA in the hippocampus of patients with mild cognitive impairment. Using similar methods, a recent structural connectivity study found that the cognitively normal elder adults showed increased connectivity along with an increased tau deposition in the bilateral hippocampus, whereas the AD‐spectrum group in these regions showed decreased connectivity (Shigemoto et al., 2018). However, these studies all considered the hippocampus as an entity, without dissecting into the intra‐hippocampal patterns. The present study performed the MRI‐histology correlation analysis in a voxel‐wise manner in each hippocampal layer, and found that with Aβ accumulation, the FA in SL layer and the MD in SR layer increased, and the ISO in SM layer decreased in AD. These findings suggest that Aβ protein may have a selective effect on hippocampal layers in AD. Its deposition impaired the hippocampal microstructure (e.g., increased diffusivity in SR), but also seem to induce a compensatory response (e.g., increased anisotropy in SL and decreased diffusivity in SM) against neuronal injury or chronic inflammation (Shigemoto et al., 2018). Notably, the SRLM layer was often reported to show significant atrophy in AD patients in in vivo MRI studies (Boutet et al., 2014; Xie et al., 2020). Here our findings in the three layers may provide supportive microstructural and molecular evidence for such a morphologic change at the macroscale.

Interestingly, we found the SP layer showed significant negative correlations between the Tau deposition and nQA as well as ISO. Our recent study also pointed that the magnetic susceptibility and T2* in the SP layer was correlated with the tau content (Zhao et al., 2021). SP layer is known to have rich pyramidal cells, in contrast to the other axon‐rich layers in the CA subfield (Van Hoesen & Hyman, 1990). Therefore, our results that the tau contents primarily affected the SP layer, while the Aβ contents correlated with the changes in SM/SL/SR layers in AD, supported our previous conclusion that the tau‐pathology may favorably affect the cellular structures and the Aβ‐pathology may have a larger impact on the axonal processes (Zhao et al., 2021). It is also worth noting that the correlation between tau content and nQA and ISO was not seen in PART patients, indicating that tauopathy in AD and PART may be different, and further investigation is clearly needed in future studies when more specimen are available.

Additionally, it is well known that hippocampal atrophy has been used to assist the clinical diagnosis of AD (Albert et al., 2011). Studies based on dMRI have demonstrated that DTI‐derived FA and MD values of hippocampus are useful biomarkers for AD whereas MD may be a more sensitive marker than FA to discriminate subjects with mild cognitive impairment from healthy subjects (Kehoe et al., 2014; Palesi et al., 2012). In addition, GQI‐derived diffusion characteristics of the hippocampus may be a potential early marker for loss of medial temporal lobe connectivity for AD (Perea et al., 2018). Existing lines of evidence indicate that MD of the anterior hippocampus is more predictive than ordinary volumetric indices for the degree of episodic memory impairment in patients with early AD (Fellgiebel & Yakushev, 2011). Moreover, the lesion of intra‐hippocampal fiber connection, such as Perforant path, results in neuronal loss of hippocampal layer, which is one of the earliest hallmark features of AD (Márquez & Yassa, 2019). Our recent study found that T2* measurement showed a decrease in all layers of anterior hippocampus from HC to PART to AD (Zhao et al., 2021). The present study demonstrated a trend from HC, PART to AD in the variances of the projection density between the hippocampal layers, suggesting the intra‐hippocampal connection changes may reflect the early disease progression of AD. We found that the dMRI measurements in several hippocampal layers were significantly correlated with the deposition of Aβ or tau protein. This indicates that the microstructure changes in cell layers within the hippocampus may be used as a biomarker for the early detection of AD.

### 4.4 Limitations

Several limitations need to be noted in the present study. First, our sample size was too small for statistical comparisons between groups, and thereby, we used the variance analysis that has been used in the previous ex vivo studies with small sample size (Antharam et al., 2012; Ke et al., 2020). Second, we only focused on the anterior part of hippocampus but not the entire hippocampus. Thus, the findings in the current study may not be generalized to the middle or tail parts of hippocampus. Third, we did not use advanced dMRI models (Crombe et al., 2018) to examine axonal density, myelination and fiber coherence, etc. Therefore, we may not answer which microstructural changes contribute to the pathology in AD. Fourth, previous studies have demonstrated that formalin fixation affects the MR parameters. While we cannot evaluate the impact of fixation on dMRI measurements, fixation time of the three groups in the present study did not have significant differences (ANOVA: *F* = 0.135, *p* = .876), and none of the dMRI measurements showed correlation with the fixation time (*p* > .05). Finally, the MRI‐histology correlation in the present study was found in a single AD sample, and correlation coefficients were relatively weak. It is necessary to collect more samples to validate our findings in the future.

## 5 CONCLUSIONS

The present study used high‐resolution ex vivo dMRI to investigate the layer‐specific anterior hippocampal microstructure and the intra‐hippocampal pattern and pathological changes in HC, PART, and AD samples. AD individuals displayed a larger variability of dMRI measurements in hippocampal layers compared with NC and PART. Moreover, the dMRI indices in the SM, SL, SR, and SP layers were correlated the Aβ/tau contents in AD. These findings suggest that mesoscopic dMRI can resolve intra‐hippocampal microstructure, and the Aβ/tau burden in AD shows layer‐specific effect on the anterior hippocampus.

## FUNDING INFORMATION

This work was supported by the Ministry of Science and Technology of the People's Republic of China (2018YFE0114600, 2021ZD0200202), National Natural Science Foundation of China (61801424, 81971606, 91859201, 61801421, 81971605, and 81971184), and the Science and Technology Department of Zhejiang Province (202006140, 2022C03057)， Fundamental Research Funds for the Central Universities of China (2019QNA5024 and 2019FZJD005), Youth Program of National Natural Science Foundation of China (82001907).

## CONFLICTS OF INTEREST

The authors declare no potential conflict of interest.

## Supporting information


**Appendix S1** Supporting Information

## Data Availability

The data that support the findings of this study are available from the corresponding author upon request.
