# Supplementary material for: Layer‐specific microstructural patterns of anterior hippocampus in Alzheimer's disease with ex vivo diffusion MRI at 14.1 T
Source: Hum Brain Mapp. 2022 Sep 2;44(2):458–71. doi: 10.1002/hbm.26062 (PMC9842914; doi:10.1002/hbm.26062)
Supplement: Supplementary file 1 — Appendix S1 Supporting Information [file HBM-44-458-s001.docx]

**Layer-specific microstructural patterns of anterior hippocampus in Alzheimer disease with *ex-vivo* diffusion MRI at 14.1 T**

**Supplementary Methods**

**Immunohistochemistry**

The brain tissue sections were dewaxed in xylene, rehydrated through the decreasing concentration series of ethanol, and washed in 1× phosphate-buffered saline (PBS). Then, slices were blocked with 3% H_2_O_2_ for 10 min and immerged in boiling citrate buffer (pH 6.0) for 15 mins, and washed twice in PBS. The slices were immunolabeled with the primary antibodies (AT8 or Aβ as listed in Table S1) that were diluted in blocking buffer (1× PBS+0.1% Triton +3% normal goat serum) and incubated at 4°C overnight. After being transported to 37 °C for 60 mins, slices were treated with a secondary antibody（goat anti-mouse IgG) at 37 °C for 60 mins and washed in 1× PBS. The slices were immersed with 3, 3’-diaminobenzidin (DAB), kept at room temperature for 3 mins. Finally, the sections were dehydrated, cleared and mounted with neutral gums.

**Table S1 Primary antibodies used for immunohistochemistry staining.**

| **Primary Antibodies** | **Abbreviation** | **Host** | **Dilution** | **Vendor** |
| --- | --- | --- | --- | --- |
| Anti-Hyperphosphorylated-tau | AT8 | Mouse | 1:200 | Thermo Fisher Scientific |
| Anti-amyloid β protein | Aβ | Mouse | 1:200 | Sigma-Aldrich |

**Supplementary Results**

**Impact of aging on hippocampal MRI measurements**

The volumes in the SM, SR, and SO layers were negatively correlated with age in the 10 hippocampal specimen (SM/SR/SO: r = -0.69/-0.67-0.76, p = 0.03/0.03/0.01, FDR adjusted 0.05 < p < 0.1) (Figure S1 A). The streamline density in the SO layer exhibited a negative correlation with age (r = -0.84, p = 0.002, adjusted p < 0.05) (Figure S1 B). Strong positive correlations (r > 0.85, p < 0.0001, adjusted p < 0.05) for volumetric change were evident between CA layers (SO/SP/SL/SR), as well as between DG layers (SM/SG/PO) (pink boxes in Figure S1 C). However, there were no significant inter-subfields correlations (p > 0.05). This pattern was also seen in streamline density-based correlation for within-subfields correlation (r > 0.90, p < 0.0001, adjusted p < 0.05) (pink boxes in Figure S1 D); additionally, significant inter-subfield connections were found between PO/SG/SM and SL/SP/SR (r > 0.75, 0.001 < p < 0.01, adjusted p < 0.05, white boxes in Figure S1 D).


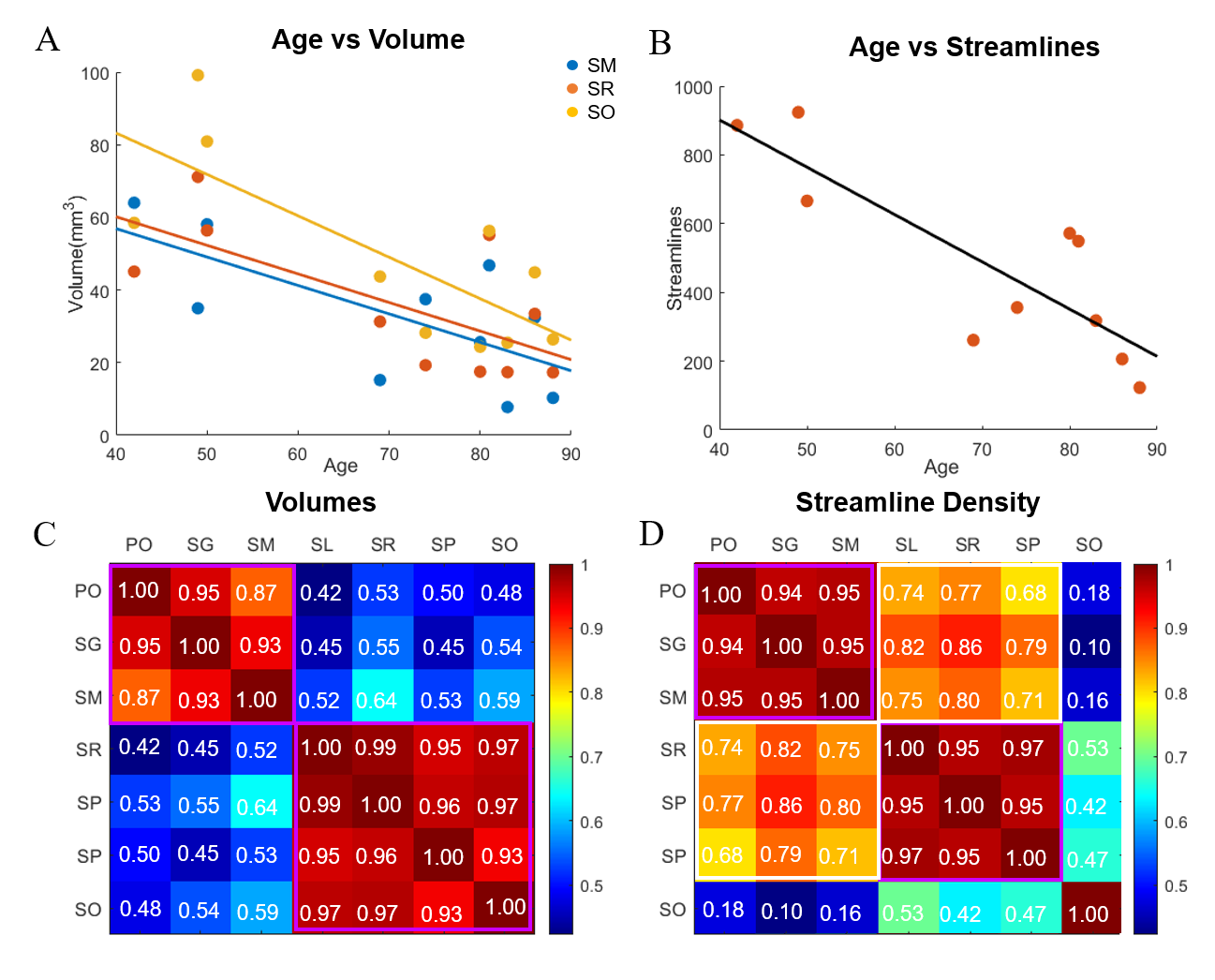


**Figure S1. Age effects on dMRI measurements.** Age is negatively correlated with volumes of SM, SR and SO layers (A), and the number of streamlines in SO layer (B). (C) The correlation matrix between layers in terms of their age-dependent volume change. Colormaps indicated the correlation coefficients (r) and the exact r values were labeled. Strong positive correlations were found among the layers within the CA1 or DG subfield (pink boxes), but no significant correlation was observed between the subfields. (D) Correlations of age-dependent streamline density change between the layers. Except for the within-subfield correlations (pink boxes), significant inter-subfield correlations (white boxes) were also found


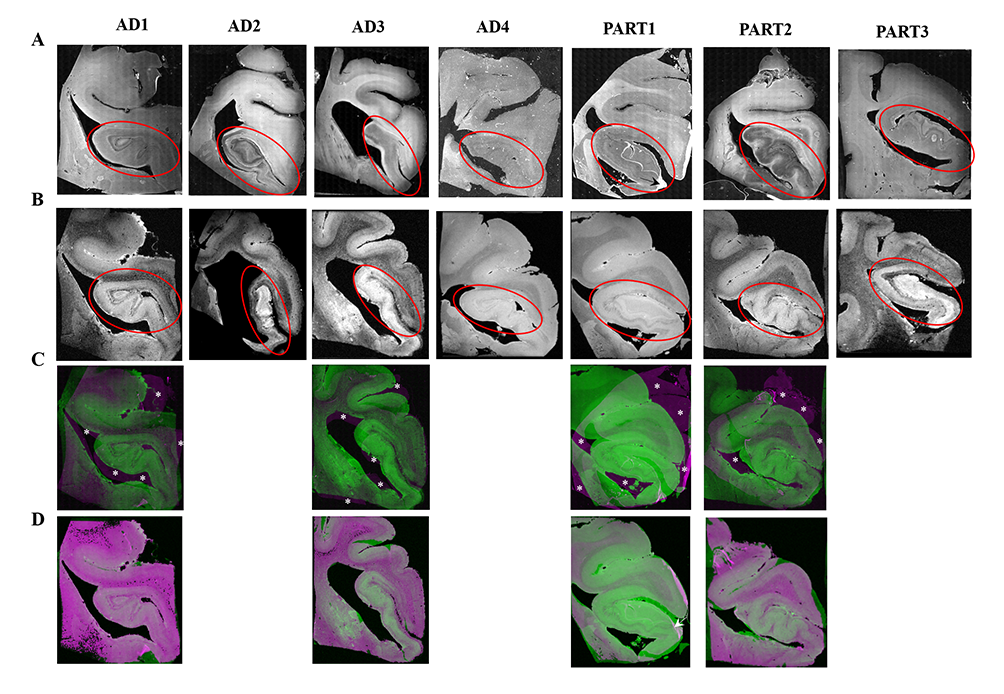


**Figure S2. The registration between MRI (template) and histological (target) images (Tau staining).** (A) and (B) show the normalized histological image and the most similar MRI slice, respectively. The areas with red circles represent the hippocampus; (C) shows the histological images (magenta) overlaid on the MRI image (green) before registration; the areas with white stars represent the misregistered areas between two images; (D) shows the registered histological images (magenta) overlaid on the MRI image (green) after registration. The hippocampal slice of PART1 is slightly folded in the corner (black arrow), leading to a registration error (white arrow) between MRI and histology.


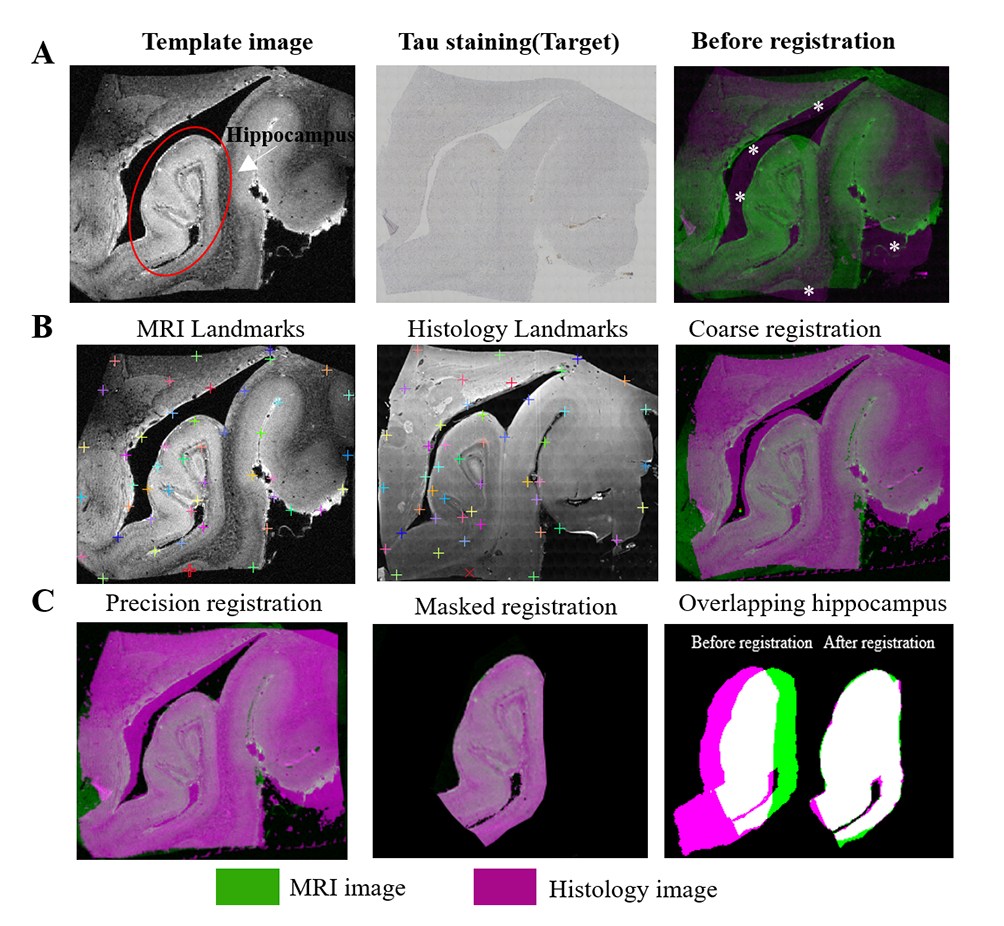


**Figure S3. The registration processing between MRI (template) and histological (target) images (Tau staining) of AD1 case.** (A) shows the original histological image, the corresponding MRI slice and their overlapping image. The area with red circle represent the hippocampus, and those with white stars represent the regions showing the most evident differences between two images before the registration; (B) shows the landmarks on MRI and histology images, and the overlapping image of template and target images after landmark-based registration; (C) shows the histological images overlaid on the MRI image after all registrations, the overlapping image within a hippocampal mask and the hippocampal histological images before and after registration overlaid on the MRI image. Magenta and green represent MRI and Histology image in all overlapping image, respectively.


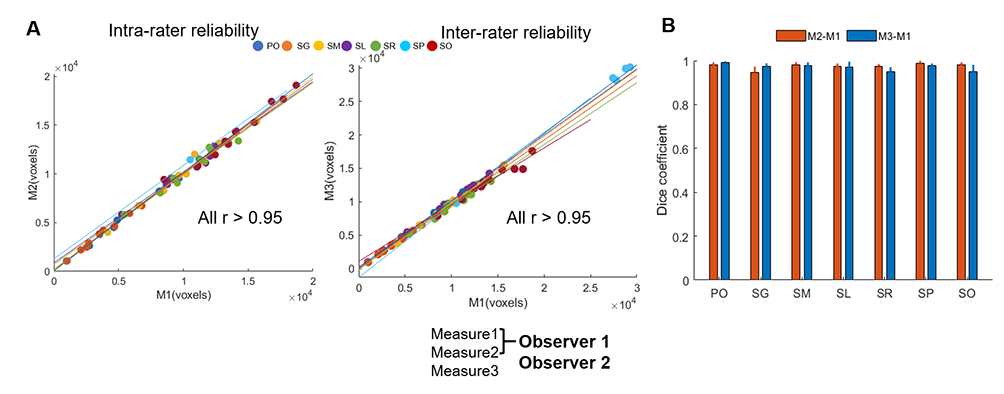


**Figure S4. The reliability of manual segmentation for hippocampal layers.** (A) shows a very high (r > 0.95) inter- and intra-rater reliability for all hippocampal layers; (B) shows a very high similarity (dice index > 0.95) for intra- and inter-rater segmentations in all hippocampal layers. M1, M2 and M3 represent the measure 1,2 and 3 from observer 1 and observer 2, respectively. Note that we selected 10 slices uniformly from MR image of each anterior hippocampal sample to perform the delineation of seven hippocampal layer by two observers (L.W. and Z.Z.), in order to evaluate reliability of manual segmentations.


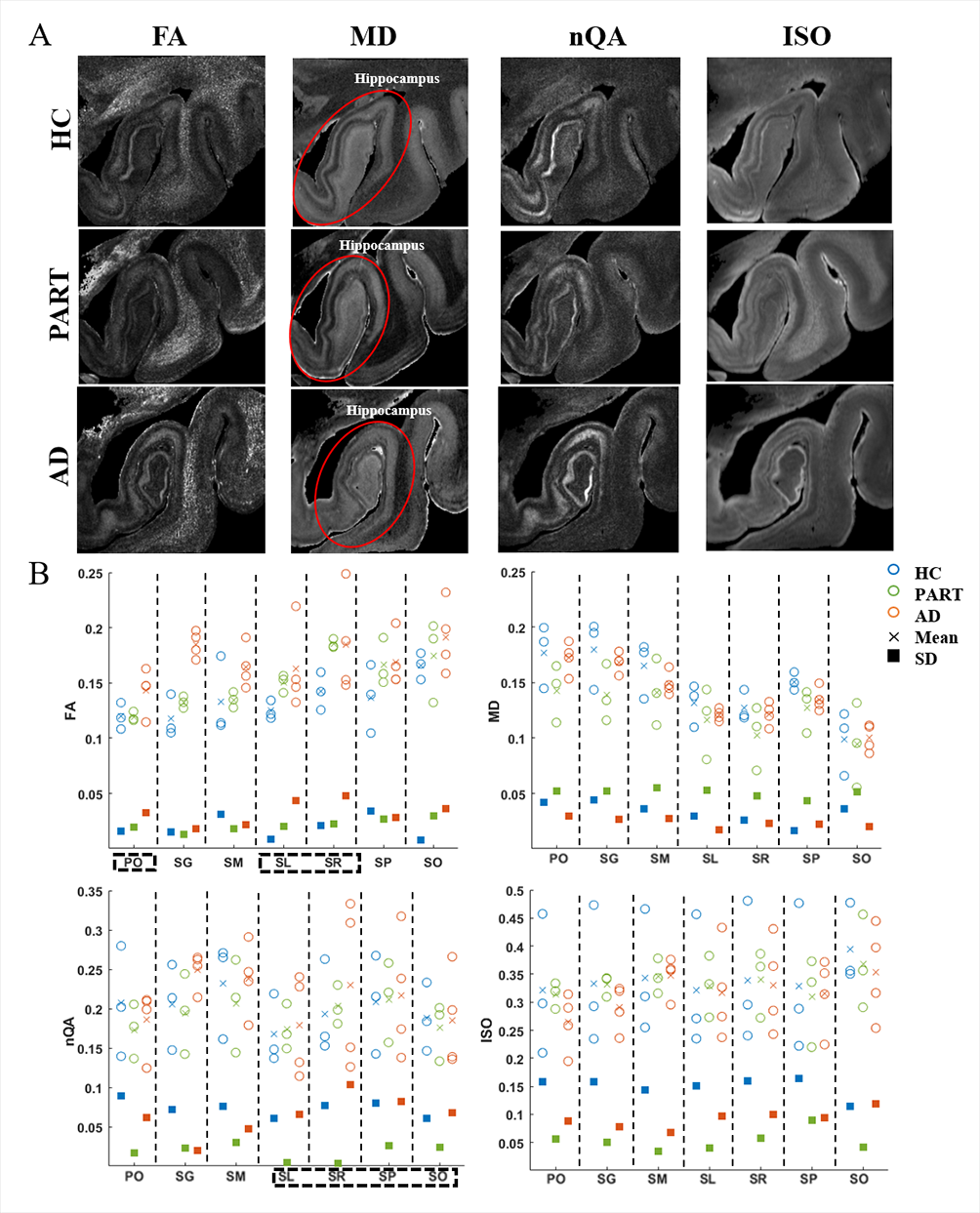


**Figure S5.** **Comparisons of the dMRI-based microstructural indices among three groups.** (A) Coronal view of the FA, MD, nQA and ISO maps of a control, PART and AD hippocampus. (B) Layer-specific patterns of MD, FA, nQA and ISO measurements in all samples. Each circle represents an individual sample; blue, green and red represent the HC, PART and AD group, respectively; fork and square markers are the group mean and standard deviation, respectively. Variabilities of FA and nQA measurements were both higher in the SL and SR layers of the AD samples than those of HC and PART (outlined by black rectangles). Note that the standard deviation represents the variability of residuals for each dMRI measurement after correcting the hippocampal volume and age.


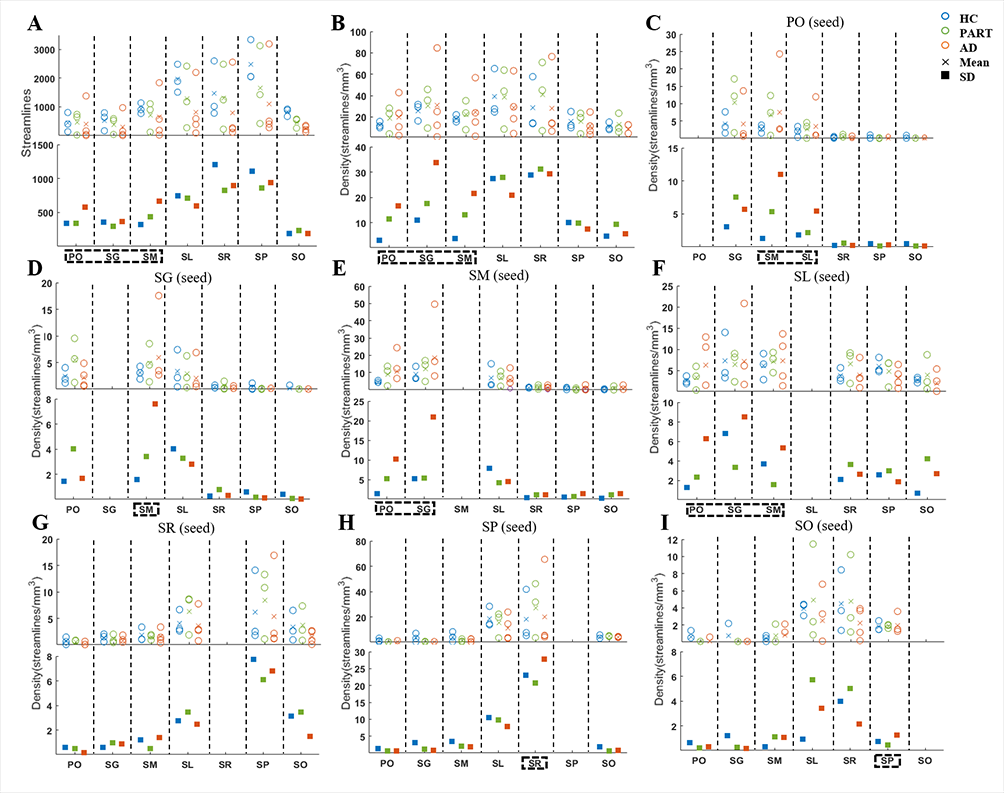


**Figure S6. Layer-specific patterns of the number of streamline and streamline density in each hippocampal layer (A-B) and the density from each of the seed layer to the other layers (C-I) for all samples.** Variabilities in the connections from three DG layers to the other layers were higher in the AD samples than those of HC and PART (black rectangle). Note that the standard deviation represents the variability of residuals for each dMRI measurement after correcting the hippocampal volume and age.

**Supplementary Discussion**

**Effect of aging on hippocampal layers**

Although most neuroimaging studies in AD have found that regional atrophy is primarily located in CA1, the selective regional vulnerability in the hippocampal subfields during healthy aging is controversial in the previous cross-sectional studies (De et al., 2015). An ex-vivo MRI study found that age was negatively associated with the volumes of DG and CA1 but not the SRLM (a set of the SL, SR and SM layers)(Adler et al., 2018). A recent in-vivo study reported that the DG was the most vulnerable subfield to the effects of aging while the CA was the primary target of AD pathophysiological processes (Nadal et al., 2020). These findings suggest that the volume changes with age in different hippocampal subfields/layers may be affected by aging, AD or both. Moreover, DTI-based studies found a positive effect of age on MD in the whole hippocampus, CA2-3 and fimbria as well as a negative effect of age on FA in the subiculum (Pereira et al., 2014; Wolf et al., 2015). In addition, a previous study in healthy older adults using structural and dMRI demonstrated the age effect on hippocampal volume was most prominent in anterior part of the hippocampal head, whereas age effect on hippocampal microstructural integrity was mainly found in the hippocampal body and tail (Wolf et al., 2015). Considering that the hippocampal layers used in the current study all located in the hippocampal head, the age effect on volume is expected to be more prominent than dMRI measurements, which is supported by our results (Figure. S1 A-B). Collectively, the age correlation analysis indicate that the hippocampal morphology and microstructural changes provide complementary information about the age-related processes, and such processes exhibited clear layer specificity. Note that the age correlation was performed over HC, PART and AD samples, and therefore, the relation reflected a mixed effect of aging and pathology.

**Reference**

Adler, D. H., Wisse, L. E., Ittyerah, R., Pluta, J. B., Ding, S.-L., Xie, L., . . . Schuck, T. (2018). Characterizing the human hippocampus in aging and Alzheimer’s disease using a computational atlas derived from ex vivo MRI and histology. *Proceedings of the National Academy of Sciences, 115*(16), 4252-4257.

De Flores, R., La Joie, R., & Chételat, G. (2015). Structural imaging of hippocampal subfields in healthy aging and Alzheimer’s disease. *Neuroscience, 309*, 29-50.

Nadal, L., Coupé, P., Helmer, C., Manjon, J. V., Amieva, H., Tison, F., . . . Planche, V. (2020). Differential annualized rates of hippocampal subfields atrophy in aging and future Alzheimer's clinical syndrome. *Neurobiology of aging, 90*, 75-83.

Pereira, J. B., Valls‐Pedret, C., Ros, E., Palacios, E., Falcón, C., Bargalló, N., . . . Junque, C. (2014). Regional vulnerability of hippocampal subfields to aging measured by structural and diffusion MRI. *Hippocampus, 24*(4), 403-414.

Wolf, D., Fischer, F. U., de Flores, R., Chételat, G., & Fellgiebel, A. (2015). Differential associations of age with volume and microstructure of hippocampal subfields in healthy older adults. *Human brain mapping, 36*(10), 3819-3831.
